# Exploring the Potential Use of *Hylocereus polyrhizus* Peels as a Source of Cosmeceutical Sunscreen Agent for Its Antioxidant and Photoprotective Properties

**DOI:** 10.1155/2020/7520736

**Published:** 2020-05-04

**Authors:** Ramya Vijayakumar, Siti Salwa Abd Gani, Uswatun Hasanah Zaidan, Mohd Izuan Effendi Halmi, Thiruventhan Karunakaran, Mohd Razak Hamdan

**Affiliations:** ^1^Halal Products Research Institute, Universiti Putra Malaysia, Putra Infoport, 43400 UPM, Serdang, Selangor, Malaysia; ^2^Department of Agriculture Technology, Faculty of Agriculture, Universiti Putra Malaysia, 43400 UPM, Serdang, Selangor, Malaysia; ^3^Department of Biochemistry, Faculty of Biotechnology and Biomolecular Sciences, Universiti Putra Malaysia, 43400 UPM, Serdang, Selangor, Malaysia; ^4^Department of Land Management, Faculty of Agriculture, Universiti Putra Malaysia, 43400 UPM, Serdang, Selangor, Malaysia; ^5^Centre for Drug Research, Universiti Sains Malaysia, 11800 USM, George Town, Penang, Malaysia; ^6^School of Chemical Sciences, Universiti Sains Malaysia, 11800 USM, George Town, Penang, Malaysia

## Abstract

Currently, consumers' demand for sunscreens derived from natural sources that provide photoprotection from ultraviolet (UV) radiation is pushing the cosmetic industry to develop breakthrough formulations of sun protection products by incorporating plant antioxidants as their active ingredients. In this context, the present study was initiated to evaluate the antioxidant and photoprotective properties of the underutilized *Hylocereus polyrhizus* peel extract (HPPE) using *in vitro* spectrophotometric techniques. The phytochemical screenings of HPPE conducted via high-performance liquid chromatography (HPLC) and ultra-high-performance liquid chromatography-quadrupole time-of-flight mass spectrometry (UPLC-QTOF/MS) revealed the presence of phenolic acids and flavonoids as the major secondary metabolites in HPPE. The antioxidant potentials evaluated based on 2, 2′-azino-bis(3-ethylbenzothiazoline-6-sulfonic acid) (ABTS) radical and total antioxidant capacity assays were in the range of 22.16 ± 0.24%–84.67 ± 0.03% with 50% inhibitory concentration (IC_50_) of 36.39 ± 0.04 *μ*g/mL and 23.76 ± 0.14%–31.87 ± 0.26% (IC_50_ = 21.93 ± 0.07 *μ*g/mL), respectively. For the photoprotective evaluation, the results showed that HPPE had significantly high absorbance values (3.1–3.6) at 290–320 nm with an exceptional sun protection factor (SPF) value of 35.02 ± 0.39 at 1.00 mg/mL. HPPE also possessed a broad-spectrum shielding power against both UVA and UVB radiations. Hence, in terms of practical implications, our findings would offer an exciting avenue to develop a photoprotective formulation incorporating the ethanolic extract of *Hylocereus polyrhizus* peels as a synergistic active ingredient for its excellent UV absorption properties and the strong antioxidant activities.

## 1. Introduction

Belonging to the *Hylocereus* genus, pitaya or more commonly known as dragon fruit is a climbing vine cactus species that have successfully attained international recognition, both as an ornamental plant and as an economical fruit crop [1, 2]. Regarded as an outstanding source of natural antioxidants and micronutrients [3–8], this superfruit is not only being consumed fresh but also being transformed into major ingredients for many innovative food products that correspond to consumers' quest [9]. In Malaysia, following the overwhelming demand from both local and international markets, the cultivation of red pitaya, *Hylocereus polyrhizus* (*H. polyrhizus*), is hiking substantially for the past few years [10]. Moreover, *H. polyrhizus* is perceived to be the consumers' favourite owing to its sweetness compared to the other types of pitayas [11]. Unfortunately, the tremendous increment in *H. polyrhizus* processing produces massive amounts of by-products especially the peels and seeds. In fact, according to the report by United Nations Food and Agriculture Organization (FAO), the waste materials originating from vegetables and fruits account for a whopping 60% when compared to other types of foods. Generally, the typical wastes of pitayas are estimated to be in the range of 30–45% [12]. As for *H. polyrhizus* in particular, the peels comprise approximately 22–44% while the composition of seeds being discarded is only about 2–4% [13, 14]. Thus, compared to the seeds, a massive quantity of *H. polyrhizus* peel wastes are being produced and, as previously reported in the literature by Jamilah et al. [15], the peels being disposed during processing are rich sources of antioxidants, vitamins, soluble and insoluble fiber, pectin, and betacyanin pigment. In addition, the decomposition of these waste materials generates unpleasant smell and causes detrimental effects by emitting harmful greenhouse gases [16, 17].

Regarded as the largest organ of the human body, skin acts as an indispensable protective wall that prevents the invasion of pathogens and other disease-causing microorganisms into the body's internal system. Everybody, regardless of their age, dreams of having a healthy-looking flawless skin that is free from tan, wrinkles, hyperpigmentation, and other skin issues. However, several *in vitro*, epidemiological, and clinical research works have demonstrated that prolonged exposure to UV light from sunlight is the predominant cause for the wide range of skin disorders including dilation of blood vessels, loss of collagen, wrinkling, scaling, dryness, and, most threateningly, both the melanoma and nonmelanoma skin cancers [18]. The initiation of reactive oxygen species (ROS) formation which possesses high reactivity with DNA, proteins, and fatty acids that often leads to the deterioration of the antioxidant system due to oxidative damage is highly associated with continuous exposure to sunlight and other external factors [19]. UV radiation is the particular section of the electromagnetic spectrum between X-rays and visible light that falls between 40 and 400 nm. The solar UV radiation can be categorized as 3 types in accordance with their respective wavelengths which are UVA (320–400 nm), UVB (290–320 nm), and UVC (200–290 nm). UVA radiation has the capacity of penetrating deep, approximately 1 mm into both dermis and epidermis layers of the skin. Besides, it is also responsible for the tanning effect which causes immediate skin darkening as a result of excessive melanin secretion in the outer epidermis layer. This phenomenon leads to serious consequences such as suppression of immunologic functions, premature photo-aging, and endothelial cell necrosis which in turn damages the dermal blood vessels [20]. Not only that UVA is 10 times more powerful in inducing lipid peroxidation compared to UVB [21]. Typically known as the burning rays, UVB is 1000 times more capable of causing sunburn in comparison to UVA and sunburned skin is the major contributor for skin cancer. The basal epidermal cell layer of the skin is the main target area for UVB but it is much more genotoxic than UVA radiations. The UVC radiations despite being filtered by the stratospheric ozone layer also carry the risk of skin cancer as they trigger the generation of ROS which results in collagen breakdown, procollagen production, and oxidative stress [20].

Skin hypersensitivity is the main concern for people with sensitive skin when it comes to opting for chemical sunscreens due to the potential risks that these unknown chemicals might trigger. Despite the fact that various hypoallergenic cosmetic products have been introduced in the market to cater to the needs of customers with sensitive skin, the options available particularly for sunscreens products are still scanty. With regard to this situation, research studies are now being focused on developing herbal sunscreens by incorporating naturally derived plant bioactive which are more efficacious with minimal or no adverse effects [22]. In addition, artificial sunscreens also cause other serious implications as there are claims that high levels of synthetic sunscreen ingredients in the blood of expecting mothers might result in underweight babies [23]. Not only the occlusiveness and opaque properties of inorganic sunscreens make them cosmetically undesirable, but their active ingredients have also been progressively reported for causing various health problems such as allergic and contact dermatitis, contact urticarial, photoallergic, and phototoxic reactions, and even solitary development of acute anaphylactic reactions [24]. In contrary, herbal sunscreens work efficiently even after a chronic exposure and have the capabilities of hindering the formation of skin cancer by interrupting multiple pathways which lead to carcinogenesis [25, 26]. Phytochemicals derived naturally from plant sources are attaining increasing amount of attention worldwide especially in the pharmaceutical and cosmetic industries to be used as photoprotective agents to prevent various skin related problems induced primarily by sun exposure [18]. In fact, the wide array of antioxidant constituents retrieved from multiple parts of the plants possesses great potentials to inhibit the molecular damage by scavenging and destroying the ROS generated from biochemical pathways, pollution, smoking, and drugs. In the context of dermatology, polyphenols are the most important class of natural products as they are distinguished by an absorption spectrum that can filter the UV radiations efficiently, thus lowering the possibilities for the radiation to penetrate deep into the dermal layers [18]. Although *H. polyrhizus* by-products especially their peels are abundant and rich in bioactive compounds which are on par, if not better than their edible counterparts, very limited studies have been conducted on *H. polyrhizus* peels to evaluate their photoprotective properties. Besides, massive quantities of solid wastes being generated every year by the food processing industry contain significant amounts of biodegradable organic substances and their disposal led to severe environmental issues. In many instances, there is still significant scantiness of pertinent feasibility studies on the exploitation of such wastes and consequently their utilization in the cosmetic, pharmaceutical, food, and beverage industries is not being wisely explored [27]. In the light of this, research focusing on the HPPE was conducted to investigate its antioxidant capacities as well as photoprotective potentials to evaluate its suitability to be utilized as the primary active ingredient in the formulation of sunscreen products.

## 2. Materials and Methods

### 2.1. Instruments and Chemicals

The chemical reagents and solvents of analytical grade used for the assays were purchased from Malaysia Sigma-Aldrich. A double-beam UV-vis (Shimadzu UV-1800) instrument was used for all the spectrophotometric measurements.

### 2.2. Preparation of *H. polyrhizus* Peels

*H. polyrhizus* fruits were purchased from Multi-Rich Pitaya Dragon Fruit Farm situated in Sepang, Selangor, Malaysia, with a latitude of 2.6875° N and a longitude of 101.7421°E, respectively. A botanist from the Biodiversity Unit, Institute of Biosciences, Universiti Putra Malaysia, identified the sample and the voucher number SK2440/16 was deposited. The fruits were washed thoroughly to remove dirt and peeled to separate the pulp from the peels which were then left for sun drying at an ambient air temperature of about 30°C from 8 am to 5 pm. Upon thorough drying, the peels were ground into a fine powder, packed in an airtight polyethylene bag, and stored in dark at room temperature for further assays.

### 2.3. Preparation of HPPE

For the preparation of extract from *H. polyrhizus* peels, 20 g of powdered peels was added to 200 mL of 82% ethanol and refluxed for 103 min at 56°C. The mixture produced was then filtered using Whatman No. 1 filter paper and the resulting supernatant was concentrated using a rotatory evaporator (EYELA, N- N series, Tokyo, Japan) at 40°C. The HPPE was then kept in the dark under refrigerated condition until further analyses [28].

### 2.4. High-Performance Liquid Chromatography (HPLC) Screening

All analyses were performed on an Agilent 1200 Series HPLC system (Waters, Milford, MA, USA) equipped with a photodiode array detector. The analytical column used was an Agilent Zorbax Eclipse Plus C18 with a length of 4.6 × 150 mm, 3.5 micron with a mobile phase consisting of mixture of solvents A (deionized water and 0.1% formic acid) and B (acetonitrile), and employing gradient elution as shown in Table 1 at a flow rate of 0.750 mL/min. The column temperature was maintained at 40°C, and the UV detection wavelength was set at 254 nm for standards, namely, rutin, apigenin, quercetin, and kaempferol, respectively. The sample injection volume was 20 *μ*L with a total run time of 18 min [29].

### 2.5. Ultra-High-Performance Liquid Chromatography-Quadrupole Time-of-Flight Mass Spectrometry (UPLC-QTOF/MS) Analysis

The chromatographic separation of phytochemicals in HPPE was conducted via ultra-high-performance liquid chromatography (UPLC-MS). The analysis was completed with a Waters ACQUITY ultra-performance LC system (Waters, Milford, MA, USA). Chromatographic separation was done using a column (ACQUITY UPLC HSS T3, 100 mm × 2.1 mm × 1.8 m, Waters, Manchester, UK). The UPLC systems were connected to Vion IMS QTOF detector (Waters, Milford, MA, USA). The mobile phases used were 0.1% formic acid (A) and acetonitrile (B). The mobile phase composition consisted of the following multistep linear gradient: 0 min, 1% B and 99% A; 0.5 min, 1% B and 99% A; 16.00 min, 35% B and 65% A; 18.00 min, 100% B and 0% A; and 20.00 min, 1% B and 99% A, respectively. The injection volume of the sample was 1 *μ*L. The flow rate was set at 0.6 mL/min. The data were obtained in the range of mass-to-charge ratio (*m/z*) 50–1500 at 0.1 s/scan in high-definition mass spectrometry elevated energy (HDMSE) with collision energies (CE) at a fixed 4 eV and ramped from 10 to 40 eV which were required for low-energy and high-energy scan, respectively.

### 2.6. Determination of Antioxidant Potentials

#### 2.6.1. 2, 2′-Azino-bis(3-Ethylbenzothiazoline-6-Sulfonic Acid) (ABTS) Radical Scavenging Assay

The ABTS radical scavenging analysis of HPPE was evaluated according to the decolourization of the ABTS radical cation (ABTS·+) [30]. First, the ABTS·+ was generated by the reaction of 10 mL of 7 mM ABTS stock solution with 10 mL of 2.45 mM potassium persulfate (K_2_S_2_O_8_) and allowed to stand in a dark place at ambient temperature for 4 to 16 h until the reaction was complete. Next, the generated ABTS·+ solution was diluted with ethanol to obtain an absorbance of 0.70 ± 0.02 at 734 nm for further measurements (Microplate Reader Infinite M200, Tecan). Then, 50 *μ*L of each extract (62.5–1000 *μ*g/mL) was mixed with the generated ABTS·+ solution and the resulting mixture was vortexed and allowed to stand in dark for 15 min. Finally, the absorbance of reaction mixtures was recorded at 734 nm spectrophotometrically. The scavenging activities of the extract and standard solution of ascorbic acid were expressed as the percentage of ABTS·+ scavenging by using

(1) $$\begin{array}{c} \%\text{ ABTS radical scavenging activity}=\left(\frac{\;A_{0}-A_{1}}{A_{0}}\right)\times 100, \end{array}$$

where *A*_0_ is the absorbance of the control and *A*_1_ is the absorbance of the sample extract. IC_50_ of ABTS·+ scavenging activity of the extract and ascorbic acid were determined using their respective calibration curves.

#### 2.6.2. Total Antioxidant Capacity Assay

The total antioxidant capacity assay of HPPE was carried out based on the phosphomolybdenum method [31]. A 0.1 mL aliquot of the sample solution (62.5–1000 *μ*g/mL) was shaken in an Eppendorf tube with 1 mL of reagent solution (600 mM sulfuric acid, 28 mM sodium phosphate, and 4 mM ammonium molybdate). Then, the reaction mixture was incubated in a water bath at 95°C for a total of 90 min. After the samples had cooled to room temperature, the absorbance of each extract was measured at 695 nm against blank. A standard of ascorbic acid was used in this assay. The total antioxidant capacities of the extract and standard solution of ascorbic acid were calculated using (1) while the IC_50_ value of the extract and ascorbic acid was determined using their respective calibration curves.

### 2.7. Sun Protection Factor (SPF) Value Determination

The maximum absorption wavelength (*λ*_max_) and SPF value were determined by a method adapted from Violante et al. with slight modifications [32]. Firstly, HPPE was diluted in absolute ethanol, obtaining different concentrations of 0.05, 0.10, 0.25, 0.50, and 1.00 mg/mL. This was followed by spectrophotometric scanning at wavelengths between 260 and 400 nm, with intervals of 5 nm. The readings were performed using 1 cm quartz cell, and ethanol was used as a blank. Zinc oxide and two commercially available sunscreen products SC1 and SC2 were used as reference substances. Calculation of SPF values was done according to the equation developed by Mansur et al. [33] as shown in

(2) $$\begin{array}{c} {\text{SPF }}_{\mathrm{spectrophotometric}}=\text{CF}\times \sum_{290}^{320}\text{EE}\left(\lambda \right)\times I\left(\lambda \right)\times \text{Abs}\left(\lambda \right), \end{array}$$

where EE (*λ*) is erythemal effect spectrum; *I* (*λ*) is solar intensity spectrum; Abs (*λ*) is absorbance of sunscreen product; CF is correction factor (=10). The values of EE × *I* are constants. These values are shown in Table 2.

### 2.8. Statistical Analysis

All assays of the ABTS radical scavenging activity, total antioxidant capacity, and SPF value determination (using different assays) were performed in triplicate. Values obtained for each sample are expressed as the mean ± standard deviation and were subjected to analysis of variance. The significance was evaluated using a one-way analysis of variance testing (ANOVA) on Expert Design software and ^*∗*^*p* < 0.05 was regarded as significant.

## 3. Results

### 3.1. High-Performance-Liquid Chromatography (HPLC) Screening

**Figure 1:**
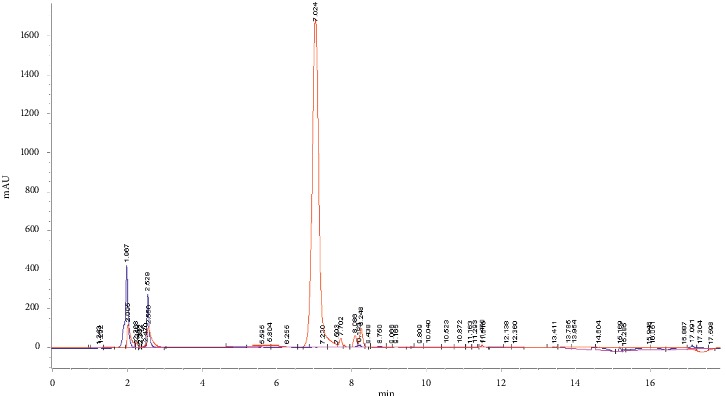
HPLC chromatogram showing the overlay of ethanolic HPPE chromatogram (blue) with rutin standard (red) using reverse phase column.

**Figure 2:**
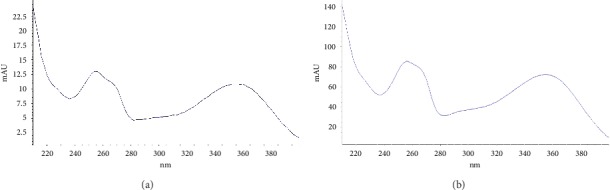
UV spectrum (in ethanol) of (a) ethanolic HPPE and (b) rutin standard.

**Figure 3:**
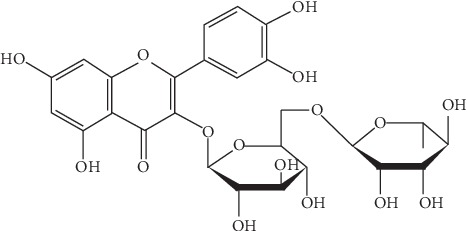
The molecular structure of rutin.

The HPLC chromatograms of all standard mixtures were recorded at 254 nm as presented in Figure 1 and the UV spectra were recorded from 220 to 380 nm as depicted in Figure 2. As illustrated in the chromatogram, all the compounds under investigation had responses at 254 nm, when they separated successfully. The phytochemical compounds under investigation were also detected based on the recorded absorption spectra and the HPLC chromatogram of the HPPE showed the presence of rutin at the retention time of 8.193 obtained under similar conditions and the corresponding UV spectra obtained online but apigenin, quercetin, and kaempferol were not detected in this extract. The molecular structure of rutin is shown in Figure 3. Rutin has been previously identified in the HPLC analysis of polyphenol compounds in the flesh and peels of *H. polyrhizus* fruit prepared *via* extract subfractionation procedure and another study which reported on the optimization of the retrieval of highly beneficial compounds from pitaya fruit waste *via* microwave-assisted extraction [35, 36].

### 3.2. Ultra-High-Performance Liquid Chromatography-Quadrupole Time-of-Flight Mass Spectrometry (UPLC-QTOF/MS) Analysis

**Figure 4:**
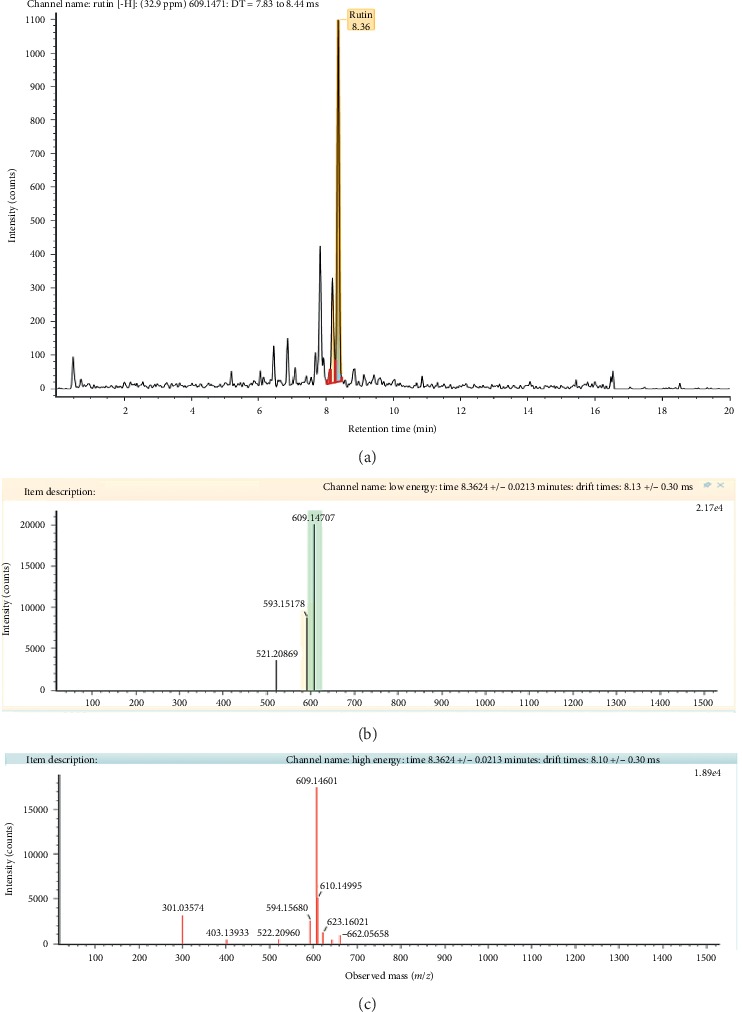
HPPE negative ion mode product of rutin compound: (a) chromatogram mass spectrum; (b) low energy of mass spectrum; (c) high energy of mass spectrum.

Separation of phytochemicals from HPPE in negative ionization mode was analyzed *via* UPLC-QTOF/MS technique and the characterized compounds are summarized in Table 3 along with their respective natural mass, observed *m/z*, and retention time. The identification of the detected compounds was carried out by comparing their retention times MS data (neutral and observed mass) and theoretical fragmentation while taking into consideration the information provided by the relevant literature and databases. The isolated compounds from HPPE were then categorized as tentative or confirmed in which the tentative category refers to the compounds that were assigned to a compound identified in the Waters library with acquisition mass accuracy less than 5 ppm and showed at least one fragment ion [37]. The confirmed category was assigned to the compound identified from the Waters library and compared with the available standard sample of the same compound. Based on the requirement set for UPLC-QTOF/MS method, nine different compounds were detected with eight of them tentatively categorized while only rutin was categorized as confirmed and validated with the standard. The HPPE negative ion mode product of rutin (chromatogram mass spectrum, low energy of mass spectrum, and high energy of mass spectrum) is shown in Figure 4. Within these analyzed compounds, six were classified under the flavonoid group while two were classified as phenolic acids (gallic acid and sinapic acid) and one vitamin B2 compound.

### 3.3. Determination of Antioxidant Potentials

#### 3.3.1. 2, 2′-Azino-bis(3-Ethylbenzothiazoline-6-Sulfonic Acid) (ABTS) Radical Scavenging Assay

**Figure 5:**
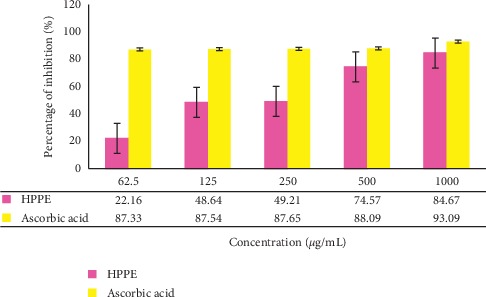
The percentage of ABTS·+ inhibition of the ethanolic HPPE and the standard solution of ascorbic acid with different concentrations (62.5–1000 *μ*g/mL). Values are the average of duplicate experiments (*n* = 3) and are represented as mean ± standard deviation. The data were significant as ^*∗*^*p* < 0.05.

The experimental data (Figure 5) revealed that the ethanolic HPPE was efficacious in scavenging the ABTS·+. The percentage inhibition of ABTS•+ exhibited by HPPE was concentration dependent whereas no significant variation among the tested concentrations of the standard solution of ascorbic acid could be observed in the scavenging of ABTS•+. At the highest concentration of 1000 µg/mL, the scavenging effect of HPPE was 84.67 ± 0.03% while ascorbic acid possessed a slightly higher value at 93.09 ± 0.21%.

IC_50_ of ABTS·+ scavenging activities in HPPE and ascorbic acid were 36.39 ± 0.04 *μ*g/mL and 86.7 ± 0.87 *μ*g/mL, respectively. The smaller the value of IC_50_, the greater the strength of the antioxidant activity. Sample which had an IC_50_ < 50 *μ*g/mL was a very strong antioxidant, 50–100 *μ*g/mL a strong antioxidant, and 101–150 *μ*g/mL a medium antioxidant, while a weak antioxidant was for IC_50_ > 150 *μ*g/mL [38]. The IC_50_ value of HPPE demonstrated that it possessed a very strong antioxidant potential when compared to ascorbic acid, emphasizing that the tested extract has an excellent ABTS·+ scavenging activity which is comparable with a potent antioxidant like ascorbic acid.

#### 3.3.2. Total Antioxidant Capacity Assay

**Figure 6:**
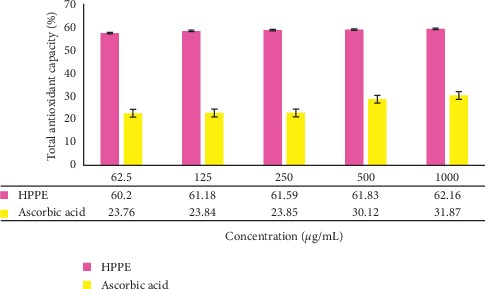
The total antioxidant capacity of the ethanolic HPPE and the standard solution of ascorbic acid with different concentrations (62.5–1000 *μ*g/mL). Values are the average of duplicate experiments (*n* = 3) and are represented as mean ± standard deviation. The data were significant as ^*∗*^*p* < 0.05.

The total antioxidant capacity of HPPE was evaluated by the phosphomolybdenum method. The principle of this assay is based on the reduction of phosphomolybdate ion with the antioxidant molecules available in the system. The reaction that follows will result in the production of greenish phosphate/MoV complex which can be evaluated spectrophotometrically [39]. The results revealed that the total antioxidant capacity of HPPE ranged from 23.76 ± 0.14% to 31.87 ± 0.26% as depicted in Figure 6. The IC_50_ values obtained for HPPE and ascorbic acid were 21.93 ± 0.07 *μ*g/mL and 13.36 ± 0.61 *μ*g/mL, respectively, depicting that both were very strong antioxidants.

### 3.4. Sun Protection Factor (SPF) Value Determination

**Figure 7:**
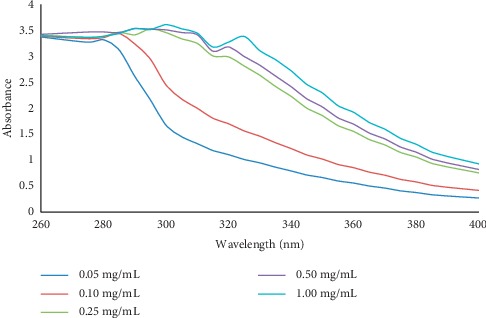
Spectroscopic absorption profile of the ethanolic HPPE at different concentrations (0.05–1.00 mg/mL) for the determination of photoprotective properties in the UVB region (260–320 nm), short UVA region (320–340 nm), and long UVA region (340–400 nm).

In this present work, HPPE with different concentrations ranging from 0.05 to 1.00 mg/mL was evaluated for its photoprotective sunscreen activity whereby the SPF values obtained were 15.38 ± 0.09, 22.62 ± 0.32, 33.38 ± 0.41, 34.35 ± 1.65, and 35.02 ± 0.39, respectively, as shown in Figure 7. The standards for comparisons, namely, zinc oxide, commercial sunscreen with titanium dioxide as an active ingredient (SC1), and commercial sunscreen with benzophenone-3 as an active ingredient (SC2), exhibited SPF values of 33.93 ± 0.08, 37.42 ± 0.64, and 15.03 ± 0.21, respectively. From the results obtained, the SPF values of both commercial sunscreens products SC1 and SC2 were surprisingly lower than their labeled SPF values. Besides, the SPF values of HPPE were very close to zinc oxide and SC1 that contains titanium oxide. According to Ratnasooriya et al., Dermatone ® which is a highly effective sun-protective agent commonly prescribed by the dermatologists contains 3% ensulizole, 7.5% octinoxate, and 9.8% zinc oxide as its active ingredient had an SPF value of 34.23 at the concentration of 2.00 mg/mL whereas HPPE was able to provide a higher SPF value at only 1.00 mg/mL [40]. Besides, as per the recommendation of skin specialists, the use of sunscreen products containing SPF values of 15 or greater is vital to lessen the harmful effects of UV rays [25]. Therefore, it can be clearly seen that this extract is a potential photoprotective agent since even at the lowest concentration of 0.05 mg/mL it possessed an SPF value of 15.38 ± 0.09.

When a sunscreen agent has a wide absorbance particularly in the wavelength of 290–320 nm, it has a better capacity in preventing sunburns which are mainly caused by UVB rays [26, 41]. At the concentration of 1.00 mg/mL, HPPE showed the highest absorbance of 3.608 at a wavelength of 300 nm which is in the UVB region. In addition, a strong absorption peak was also shown in the short-wavelength UVA region of 325 nm with an absorbance of 3.3806 as shown in Figure 7. Previous studies have demonstrated that penetration of UVB rays into the skin inclines to initiate the production of highly reactive free radicals which in turn exposes our skin to various photo-damaging effects such as wrinkling, premature aging, and even melanoma whereas UVA radiation exposes the skin to risk of excessive tanning and also encourages the onset of premature skin aging [41].

## 4. Discussion

### 4.1. High Performance Liquid Chromatography (HPLC) Screening

Rutin is basically a glycoside that incorporates flavonolic aglycone quercetin alongside disaccharide rutinose. Most early studies, as well as the current research works, have focused on exploring the pharmacological properties of rutin such as antioxidant, antiplatelet anticarcinogenic, cardioprotective, cytoprotective, antithrombotic, vasoprotective, and neuroprotective activities [42]. The distinctive polyphenol structure enables flavonoids to effectively prohibit the direct ROS scavenging activity that injures the vulnerable cells. The mode of action undertaken by rutin is through the donation of its own electrons to the free radicals such as hydroxyl radicals and superoxide radicals, to neutralize them into a more stable and nonreactive species. This action will eventually terminate the free radical chain reactions [43]. Besides, inhibition of enzymes such as xanthine oxidase and NADPH oxidase that generates ROS in rheumatoid arthritis leukocytes is another benefit of rutin [44]. In a study conducted on the rat brain cells, rutin boosted the antioxidant potentials *via* increment of copper (Cu), zinc-superoxide dismutase (Zn-SOD), catalase (CAT), and phospholipid hydroperoxide glutathione peroxidase (GSH-Px) activities and by elevating the GSH levels [45]. Other than this, by suppressing the activities of cyclooxygenases and lipoxygenases, flavonoids also decrease the proinflammatory processes in human neutrophils [46]. Not only that, rutin has been reported to display cytoprotective properties on UV irradiated fibroblast cells by reducing the amount of ROS present in the system which simultaneously aids in lowering the metalloproteinase expressions and DNA modifications [47, 48].

### 4.2. Ultra-High-Performance Liquid Chromatography-Quadrupole Time-of-Flight Mass Spectrometry (UPLC-QTOF/MS) Analysis

Although HPLC fingerprinting of HPPE did not detect quercetin and kaempferol, the derivatives of both these compounds were successfully detected *via* UPLC-QTOF/MS technique. When it comes to high sensitivity and selectivity, UPLC-QTOF/MS exhibits faster-resolving power and higher resolution performance than HPLC. In addition, the analysis of complex mixtures can be performed with ease as it requires shorter duration and the resulting peaks illustrate more information with clearer representation compared to HPLC's peaks [49]. Due to the lack of standards, only rutin was validated using standard. As illustrated in Figure 4, rutin was spotted at *m/z* 609.14 and gave rise to intense ions at *m/z* 301.03 corresponding to the loss of the rutinose unit while fragment ions at *m/z* 594.15 indicate the presence of oxygen ion, in accordance to previous literatures [50–52]. In another research that was conducted to optimize extraction conditions for higher total phenolic flavonoids recovery and to determine *in vitro* antioxidant activities of defatted *H. polyrhizus* seed extract (DPSE) using response surface methodology, UPLC-QTOF/MS analysis revealed the presence of rutin at the retention time 8.38 and *m/z* peak 609.14 which is in a good agreement with the findings of this study. Besides, sinapic acid and flavonoid compounds with core moieties of kaempferol as well as isorhamnetin were also detected in DPSE [53]. The phytochemical composition of HPPE revealed in this study also justifies the good results obtained for its total phenolic content (172.01 mg gallic acid equivalent (GAE)/g) and total flavonoid content (7.45 mg quercetin equivalents (QE)/g) that were conducted in our previous work which focused on optimizing the extraction conditions for phenolic and flavonoid compounds from *H. polyrhizus* peels using a statistical approach [28].

Phenolics and flavonoids are secondary metabolites derived from tyrosine and phenylalanine with potent antibacterial and antioxidant activities [54]. Chemically, these compounds are categorized as substances that possess an aromatic ring attached to one or more hydroxylic substituents, inclusive of their functional groups with variable structures, and these exclusive features enable them to be multifunctional [55]. The flavonoids isolated from HPPE mainly contained quercetin, kaempferol, isorhamnetin, and malvidin core moieties with their corresponding sugar moieties mostly attached to the flavonoids' basic skeletons. The quantity of sugar moieties present as well as their attachments to the respective flavonoid's structure depicts the strength of their antioxidant properties against oxidative stress induced by UV radiations and other factors. While gallic acid and sinapic acid function excellently as anti-inflammatory, anticarcinogenic, antifungal, and antioxidant agents, in addition to being a potent metalloproteinase inhibitor [56], sinapic acid also tends to show strong absorption in the UV-B region when subjected to cold, isolated environment of a supersonic expansion to explore its intrinsic UV spectral properties in detail [57]. Other than this, widely known as riboflavin, vitamin B2 was also detected in HPPE. From the context of dermatology, vitamin B2 works effectively to recycle glutathione, which is one of most pivotal antioxidants that shields the body against free radicals attack [58]. A number of authors have previously reported that riboflavin can alleviate oxidative injuries to a great extent by scavenging the free radicals and even aids in correcting pesky spots as well as revitalizing the skin by boosting cell turnover for a healthier-looking skin [59].

### 4.3. Determination of Antioxidant Potentials

Based on the results obtained for the antioxidant assays, the good antioxidant potentials exhibited by HPPE were significantly contributed by the presence of phenolic acids and flavonoids which played a pivotal part in boosting the photoprotective ability of this extract. As discussed earlier, the inclusion of sunscreen remains inevitable when it comes to protecting ourselves from the deleterious consequences of the UV radiation. Unfortunately, insufficient application, as well as compensatory exposure, is becoming major concern for sunscreen wearers who are likely to be exposed to sun longer. In reality, the degree of UV shielding power of the respective sunscreens is much weaker than depicted in the product labels. In addition, the availability of sunscreens on the current market that tends to offer more UVB than UVA protection also needs adequate attention since these formulations will be less efficient in minimizing free radicals generated *via* UVA radiations. Furthermore, the research work done by Haywood and colleagues has demonstrated that even broad-spectrum sunscreens are capable of inhibiting the generation of free radical formation by only 55% [60]. This situation necessitates the incorporation of antioxidants in the topical delivery systems in order to impart additional benefits to complement the effectiveness of UV filters. In fact, the synergism that exists between antioxidants and UV filters has been investigated in several studies. For example, Matsui et al. [61] investigated the protective benefits that will be derived *via* the combination of UV filters and antioxidants in human studies. In this particular study, the participants were given two different topical formulations: one sunscreen with an SPF value of 25 and the same sunscreen with a mixture of antioxidants, namely, caffeine, vitamin E and vitamin C, extracts of *Echinacea pallida*, gorgonian plant, and essential oil of chamomile. The experimental data obtained revealed that the group tested with sunscreen added with antioxidant mixture showed a 17% greater reduction in matrix metalloproteinase-1 (MMP-1) upon UV radiation exposure to the skin, emphasizing the crucial role played by antioxidants in photoprotection [61].

### 4.4. Sun Protection Factor (SPF) Value Determination

The sun protection factor (SPF) which was established by Sayre et al. [34] is regarded as the universal indicator employed to describe the efficiency of sunscreen products to protect the skin against sunburn [41]. It is the ratio calculated based on the energies necessary to initiate a minimal erythema dose for protected skin following an application of 2 mg/cm^2^ of sunscreen product and also the unprotected skin of human subjects, using the UV radiation produced by an artificial source. Generally, sunscreen products are categorized in accordance with their SPF values whereby values from 2 to under 12 are classified as “minimal sun protection” and 12 to under 30 are “moderate sun protection” whereas sunscreens products with SPF values of 30 and above are defined as “high sun protection,” respectively [62]. For a sunscreen product to effectively protect the skin from the deleterious effects caused by UV radiations, it must possess a wide range of absorbance which covers 290–400 nm. Moreover, higher SPF value is an indication of the efficacy of a good sunscreen product because it determines the duration up to which it protects the wearer while staying under the sun before experiencing sunburn [63].

Rutin elucidated in the HPPE has been reported in the earlier literature to possess good photoprotective properties. Rutin occupies a prominent position in the list of natural sunscreening agents, thanks to its structural arrangements that have a close resemblance with the organic UV filters as well as its exceptional antioxidant potential to scavenge free radicals triggered by UV radiations [64, 65]. Peres et al. [66] investigated the *in vitro* photoprotective efficiency, photo-stability, antioxidant properties, and *in vivo* skin tolerance which includes criteria such as skin hydration, transepidermal water loss, and erythema of a formulation comprising of rutin as its main UV filter. From the results, it was shown that the presence of rutin exhibited a positive synergic interaction with the isolated UVB filter since an increase in UVA photoprotective efficacy when there is an absence of UVA filter was witnessed [66]. In addition, the scavenging potential of rutin also seen to enhance the photo-stability of the anti-UVB system since rutin may have counteracted the degradation of other UV filters stimulated by ROS. When compared with the sample formulated solely with UVB agents, the rutin-based sample showcased excellent compatibility with the human skin and 75% higher free radical quenching ability. Although there was no favorable outcome for the prevention of the sunscreen's postirradiation photo-degradation, the sample with rutin experienced a notable improvement in the sunscreen's critical wavelength, implying its high effectiveness in the UVA region [67]. Therefore, a good photoprotection against UVA radiations is expected with the utilization of HPPE in the formulation of sunscreen products.

Besides, another experiment was performed by Gegotek et al. [68] to scrutinize the effects imposed by rutin on the proinflammatory, antioxidant, and endocannabinoid systems and proapoptotic processes of UV irradiated fibroblasts. Firstly, rutin exhibited a partially protective effect on the fibroblasts against the UVA and UVB stimulated intracellular oxidative processes and the expression of proinflammatory signaling mediators. After the UV radiation, the nuclear factor kappa-light-chain-enhancer of activated B cells (NF*κ*B) levels escalated up to 3- to 4-folds and, after treating the cells with rutin, their levels greatly decreased by 10% and 20%, respectively. Besides, tumor necrosis factor-alpha (TNF*α*) levels which showed a 4-fold increase also declined by approximately 40% after the cells were exposed to UVA and UVB radiations. Apart from lessening the translocation of NF*κ*B originating in the cytoplasm to the nucleus, rutin substantially halted the activities of enzymes attributable to the superoxide anion generation, namely, xanthine oxidase and NADPH oxidase that tend to increase in quantity after UV exposure. This is seen as an important criterion to make rutin as an efficacious sunscreen agent since the UV rays triggered the xanthine oxidase's activity by about 3- and 5-folds and more crucially an enormous increase in NADPH oxidase activities by 80% and 120%, respectively, was observed [68]. Besides, the level of superoxide anions that elevated by 4- and 5-folds increases as the UVA and UVB irradiations were successfully decreased *via* treatment with rutin by 3- and 2.5-folds, respectively. The sunburn that occurs following a prolonged sun exposure is a form of the inflammatory response induced by the immune system to initiate the healing process to replace the damaged skin cells and the findings of this study also emphasized the ability of rutin of safeguarding the fibroblast cells against inflammatory reactions mediated by UVA and UVB rays. This phenomenon which occurred in the UV irradiated fibroblast was mainly supported by the reduction in NF*κ*B levels and also of amount products such as TNF*α* synthesized *via* rutin-modulated transcriptional activity [68].

Apart from rutin, quercetin is another phytoconstituent with high photoprotective potential since both of these compounds were proven to behave in similar ways in prior works. As reported by Choquenet et al. [69], the incorporation of rutin and quercetin at a concentration of 10% (w/w) for the formulation of oil-in-water emulsions resulted in SPF values that were found to be on par to that of a standard substance widely used in sunscreens, namely, homosalate. In fact, both of these flavonoids also exhibited a nonnegligible level of photoprotection in the UVA region and when combined with another widely used UV filter, titanium dioxide, this combination gave an SPF value of 30. When compared to UVB filters which are authorized by the European Union at present, rutin and quercetin would be ranked in the ninth position (9/18), with an effectiveness comparable to homosalate that is used to establish US Food and Drug Administration standards. As far as their potency against UVA is concerned, they are also both ranked 5th out of 7 authorized filters and since their levels of protection against both UVB and UVA are alike, both rutin and quercetin render an SPF/PF-UVA ratio of less than 3. Most importantly, both of these compounds exhibited excellent photo-stabilities after 2 h of irradiation at 650 W/m^2^ [69]. Thus, the inclusion of HPPE enriched with beneficial active ingredients in the formulation of dermocosmetics provides sun protection not only on the skin's surface but also on the cellular and enzymatic levels for optimum efficacy.

## 5. Conclusions

We have demonstrated that the extract of *H. polyrhizus* peels is a potent antioxidant with excellent photoprotective properties. Besides, the phenolic and flavonoid compounds in the HPPE contributed to the overall antioxidant activities as well as the high SPF value and broad-spectrum UVA and UVB photoprotection. Based on the findings of this research, *H. polyrhizus* peels are highly competent to substitute the synthetic sunscreen agents to serve as a natural active ingredient in the remunerative cosmetic industry. The utilization of plant extracts will surely minimize the risk of various skin disorders brought about by the artificial active ingredients in commercial cosmetic products to a much greater extent. Not only that, the waste loads at the processing plant can be significantly reduced through the utilization of new or modified processing techniques or the in-plant treatment and reuse of these agricultural wastes. Further works on the development of nanoemulsion-based topical sunscreen formulation by incorporating HPPE as the main active ingredient and its *in vivo* photoprotection efficiency are currently under progress.

## Figures and Tables

**Table 1:** Analytical conditions of HPLC for analysis of the four standards.

Parameters	Conditions		
Column	Agilent Zorbax Eclipse Plus C18 (4.6 × 150 mm, 3.5 micron)		
Flow rate	0.750 mL/min		
Injection volume	20 *μ*L		
UV detection	254 nm		
Run time	18 min		

	Time (min)	% A^1^	% B^2^

Gradient	0.00	90	10
	0.50	90	10
	10.50	55	45
	12.00	30	70
	14.00	30	70
	16.00	90	10

^1^0.1% formic acid; ^2^acetonitrile.

**Table 2:** Normalized product function used in the calculation of SPF [34].

Wavelength (nm)	EE × *I* (normalized)
290	0.0150
295	0.0817
300	0.2874
305	0.3278
310	0.1864
315	0.0839
320	0.0180
Total	1.000

**Table 3:** Compounds identified in the ethanolic *Hylocereus polyrhizus* peel extract using UPLC-QTOF/MS.

No.	Component name	A	B	C	Identification status and category
(1)	Gallic acid	1.20	170.0215	169.0134	Identified, tentative
(2)	Vitamin B2	1.86	376.1383	375.1326	Identified, tentative
(3)	Quercetin-3-O-(6″-O-acetyl)-*β*-D-glucopyranoside	4.54	506.1060	505.0985	Identified, tentative
(4)	Sinapic acid	5.41	224.0685	223.062	Identified, tentative
(5)	Isorhamnetin-3-O-(2G-*α*-L-rhamnosyl)-rutinoside	5.83	770.2269	769.2189	Identified, tentative
(6)	Rutin	8.36	610.1534	609.1471	Identified, confirmed
(7)	Quercimeritrin	8.37	464.0955	463.0892	Identified, tentative
(8)	Kaempferol-3-O-*β*-D-glucopyranoside	9.60	448.1005	447.0939	Identified, tentative
(9)	Malvidin-3-O-(6-O- acetyl-*β*-D-glucopyranoside)-5-O-*β*-D-glucopyranoside	9.91	696.1902	695.1828	Identified, tentative

A: retention time (min); B: natural mass (Da); C: observed *m/z*.

## Data Availability

The data used to support the findings of this study are included within the article.
